# The effect of reducing per capita water and energy uses on renewable water resources in the water, food and energy nexus

**DOI:** 10.1038/s41598-022-11595-w

**Published:** 2022-05-09

**Authors:** Shima Kheirinejad, Omid Bozorg-Haddad, Vijay P. Singh, Hugo A. Loáiciga

**Affiliations:** 1 Department of Irrigation and Reclamation Engineering, Faculty of Agricultural Engineering and Technology, College of Agriculture and Natural Resources, University of Tehran, Karaj, Tehran, Iran; 2 Department of Biological and Agricultural Engineering and Zachry Department of Civil and Environmental Engineering, Texas A&M University, 321 Scoates Hall, 2117 TAMU, College Station, TX 77843-2117 USA; 3 Department of Geography, University of California, Santa Barbara, CA 93016-4060 USA

**Keywords:** Climate sciences, Ecology, Environmental sciences, Environmental social sciences, Hydrology, Engineering, Mathematics and computing

## Abstract

This study assesses the feedbacks between water, food, and energy nexus at the national level with a dynamic-system model, taking into account the qualitative and quantitative environmental water needs. Surface and groundwater resources are considered jointly in the water resources subsystem of this dynamic system. The developed model considers the effects of reducing the per capita use water and energy on its system’s components. Results indicate that due to feedbacks the changes in per capita uses of water and energy have indirect and direct effects. About 40% of the total water savings achieved by the per capita change policy was related to energy savings, in other words, it is an indirect saving. Implementation of per capita use reductions compensates for 9% of the decline of Iran's groundwater reservoirs (non-renewable resources in the short term) that occur during the five-year study period. The Manageable and Exploitable Renewable Water Stress Index (MRWI) corresponding to water and energy savings equals 214.5%, which is better than its value under the current situation (which is equal to 235.1%).

## Introduction

A significant percentage of the world's population does not have adequate access to water, food, and energy resources (WFE). Although efforts to achieve the UN Millennium Development Goals and later the Sustainable Development Goals (SDGs) have increased access to scarce resources, still, 25.9% of the population is affected by moderate or severe food insecurity in 2019, 2.2 billion people lacked access to potable water in 2017, and 789 million people lacked electricity service in 2018^1^. The pressure on WFE resources will increase as the world's population grows from 7.4 billion in 2016 to 9.7 billion in 2050^2^. The WFE Nexus is a concept in which reciprocity and feedbacks must be understood by policymakers to reach optimal management decisions. The concept of the WFE Nexus is examined at different scales and for different purposes, which involves diverse factors, such as the environment, climate change, and government^3^. Understanding the interrelationships that exist between the three vital resources (WFE) plays a commanding role in the quest for balancing the supply and demand of resources. There are numerous studies of the water and food nexus, which highlight the prominence of agriculture as a water consumer in most basins^4^. Studies on the water and energy nexus are in comparison less numerous^5^. Dai et al.^6^ identified five methods for studying the water and energy nexus. The focus of the water and energy nexus is on the use of energy for water extraction, distribution, desalination, transfers, and treatment, and on the use of water for electricity generation by hydropower plants, biofuels, and the exploration, extraction, and use of fossil fuels^7–10^. Dai et al.^6^ identified 15 methods that consider the environment’s role in the water and energy Nexus. Most of these 15 methods quantify the feedbacks between water and energy by considering environmental factors such as climate change and greenhouse gases. A few methods (GLEW and WATER) focus on the water and energy nexus effects on the quality and quantity of water required by the environment. As these methods and the current research method comparison shows, one neglected aspect in these two methods is the energy required to extract, purify, transfer, and distribute water resources. Besides, WATER and GLEW methods have concentrated on bioenergy and electricity, respectively, while this study considered different types of energy carriers^11–13^.

Scanlon et al.^2^ argued that research on the WFE Nexus and its impact on the terrestrial system is essential to achieve a sustainable environment. Harm to the environment jeopardizes all three WFE resources sustainability, and examining the impact of poor management in one sector on other sectors from the perspective of the WEF nexus is necessary for improving resources management and environmental protection^14^. Water, food, and energy are considered jointly in the management of reservoirs^15^. Hoff^16^ introduced the concept of WFE Nexus. Afterward, several frameworks and methods have been proposed. Several conceptual WFE Nexus frameworks that were examined on a national scale^17,18^ are listed in Table 1, and their advantages and disadvantages with respect to this paper’s model are reported. Evaluating management WFE policies at the macro (national) level with modeling scenarios is a timely endeavor. By avoiding high complexity, massive data usage, and considering regional specificity/the WFE model can be applied in different areas with minor changes. A review of the pertinent literature reveals that the concept of manageable and exploitable renewable water resources from the WFE Nexus national perspective has not been addressed in sufficient depth. Only two studies, one on a global scale and the other in the Danube River Basin, have addressed renewable water resources in the WFE Nexus framework^19,20^.

**Table 1:** Review of some of the key national-level WFE Nexus conceptual frameworks.

Analytical frameworks	Developer	Advantages over the model of this research	Weaknesses compared to the model of this research	References
WFE Nexus Rapid Appraisal Tool	FAO	Capacity to quickly assess the WFE Nexus status with a limited number of indicators; Consideration of climate change	not being able to build scenarios	Flammini et al.^45^
MuSIASEM (Multi-Scale Integrated Analysis of Societal and Ecosystem Metabolism)	Giampietro et al.	considering economic and social issues.	Model complexity and lack of user-friendly graphical interface	Giampietro et al.^46^
World Bank Climate and Disaster Risk Screening Tools	World Bank	Considering climate trends and geophysical hazards	Having Diagnostic properties and not being able to build scenarios	Dargin et al.^17^
WEF Nexus Tool 2.0	Daher and Mohtar	Considering carbon emissions	Ignoring temporal WFE nexus feedbacks; Ignoring aquatic environmental impact	Mohtar and Daher^47^; Daher and Mohtar^48^
National Outlook Model for Australia	CSIRO	Consideration of socio-economic issues, climate change and land use change	Geographically limited to Australia; imposing significant computational and input data requirements	Hatfield-Dodds et al.^49^

Studies to improve the security of the WFE components by estimating environmental water needs (simultaneously from both qualitative and quantitative perspectives) have not been sufficiently comprehensive. This work applies the concept of water footprint in the water resources subsystem of the WFE Nexus considering the qualitative and quantitative aspects of water use. Our analysis of the water resources subsystem includes the components of the hydrologic cycle at a national scale in the form of a lumped model (i.e., using average inputs and outputs nationwide), which accounts for surface water and groundwater resources. This comprehensive characterization of the water-resources subsystem of the WFE nexus improves the accuracy of the assessment of WFE interactions at the national level, and enables the calculation of manageable and exploitable renewable water resources. Also, the energy subsystem considers all types of energy carriers produced. This work’s simulation study of the WFE nexus was performed in an Anylogic software environment, which constitutes a novelty concerning WFE Nexus assessments, and this software’s capability as an optimization tool is herein demonstrated when coupled with the OptQuest tool^21^. This is the first time the Anylogic software has been applied to model the WFE Nexus. This software is superior to other similar platforms in terms of the ability to combine different types of modeling (discrete event, Agent-based and system dynamic modeling), the possibility of building scenarios, and the ease for sharing an Anylogic-based model in the Internet.

This work introduces a model that deals with the WFE Nexus at a national scale. The model is applied to Iran, which is an arid and semi-arid country and is a world power in energy production. Only a handful of comprehensive Nexus-based tools have been developed for application in Iran, and they operate at the river basin scale (see, e.g., Ravar et al.^5^; Bakhshianlamouki et al.^22^) or even smaller scales (see, e.g., Naderi et al.^23^).

## Proposed methodology and model structure

**Figure 1:**
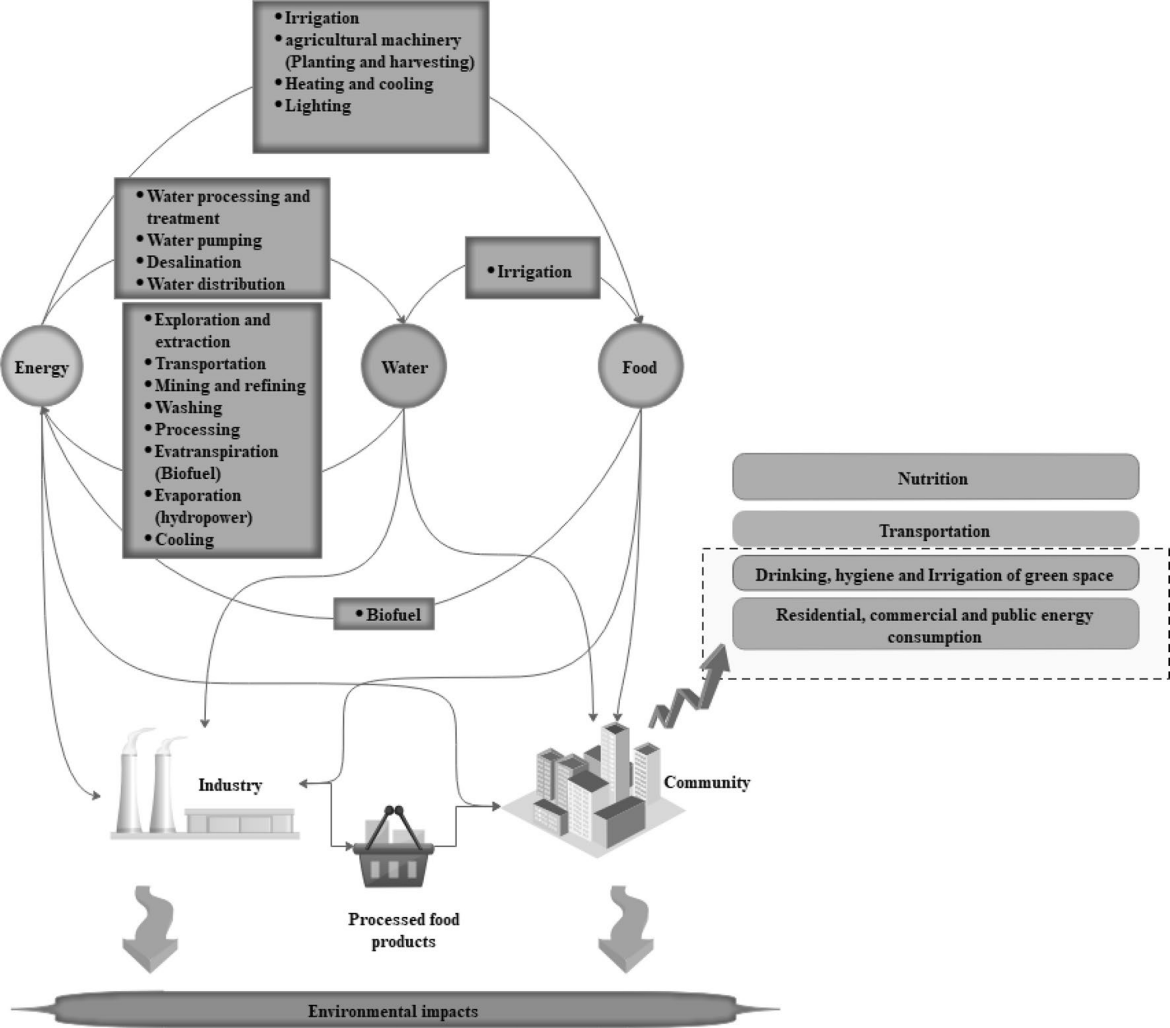
Feedbacks between resources and uses in the WFE nexus taking into account environmental considerations.

**Figure 2:**
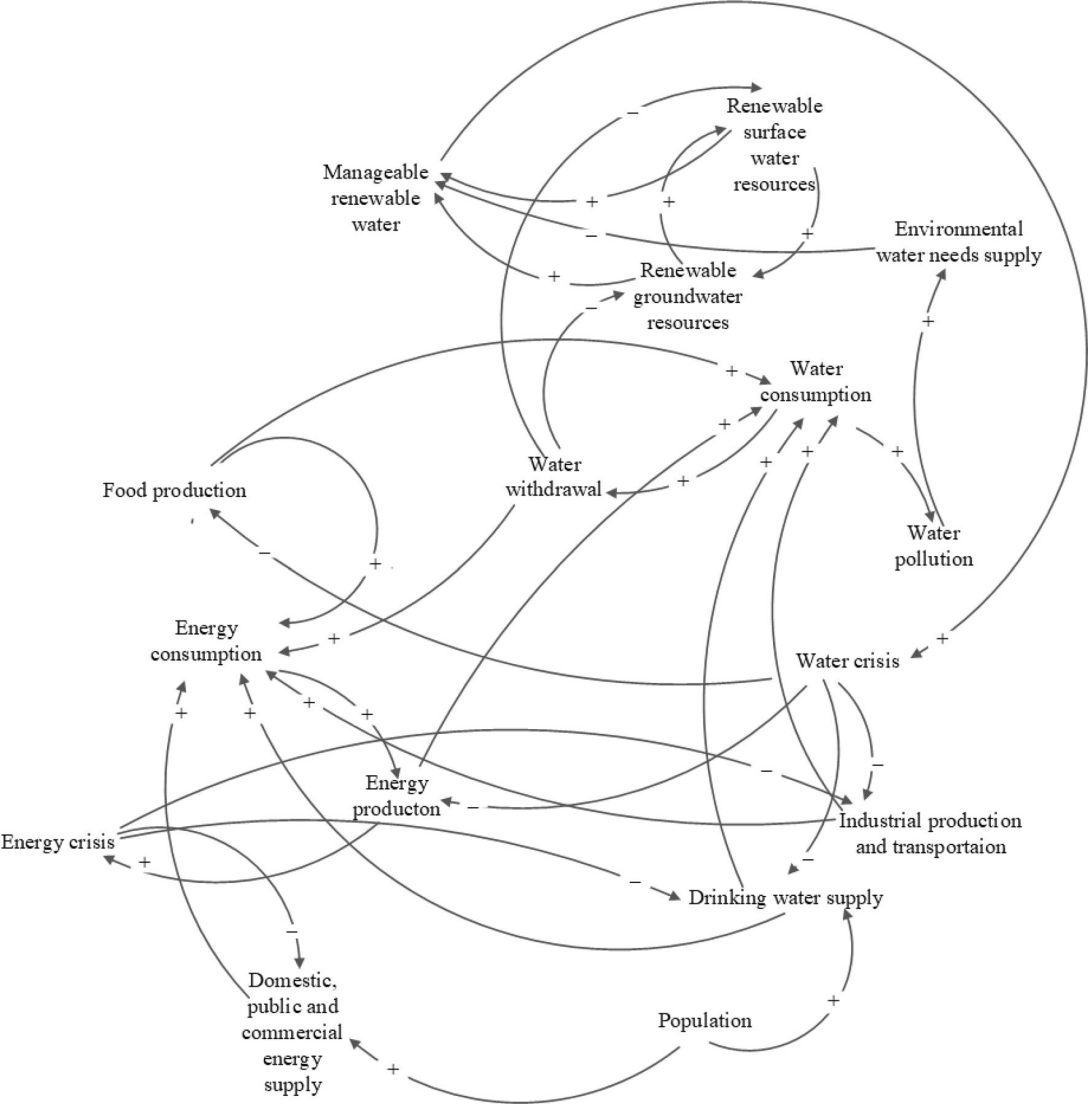
The causal loops of the model developed for simulating the WFE nexus.

**Figure 3:**
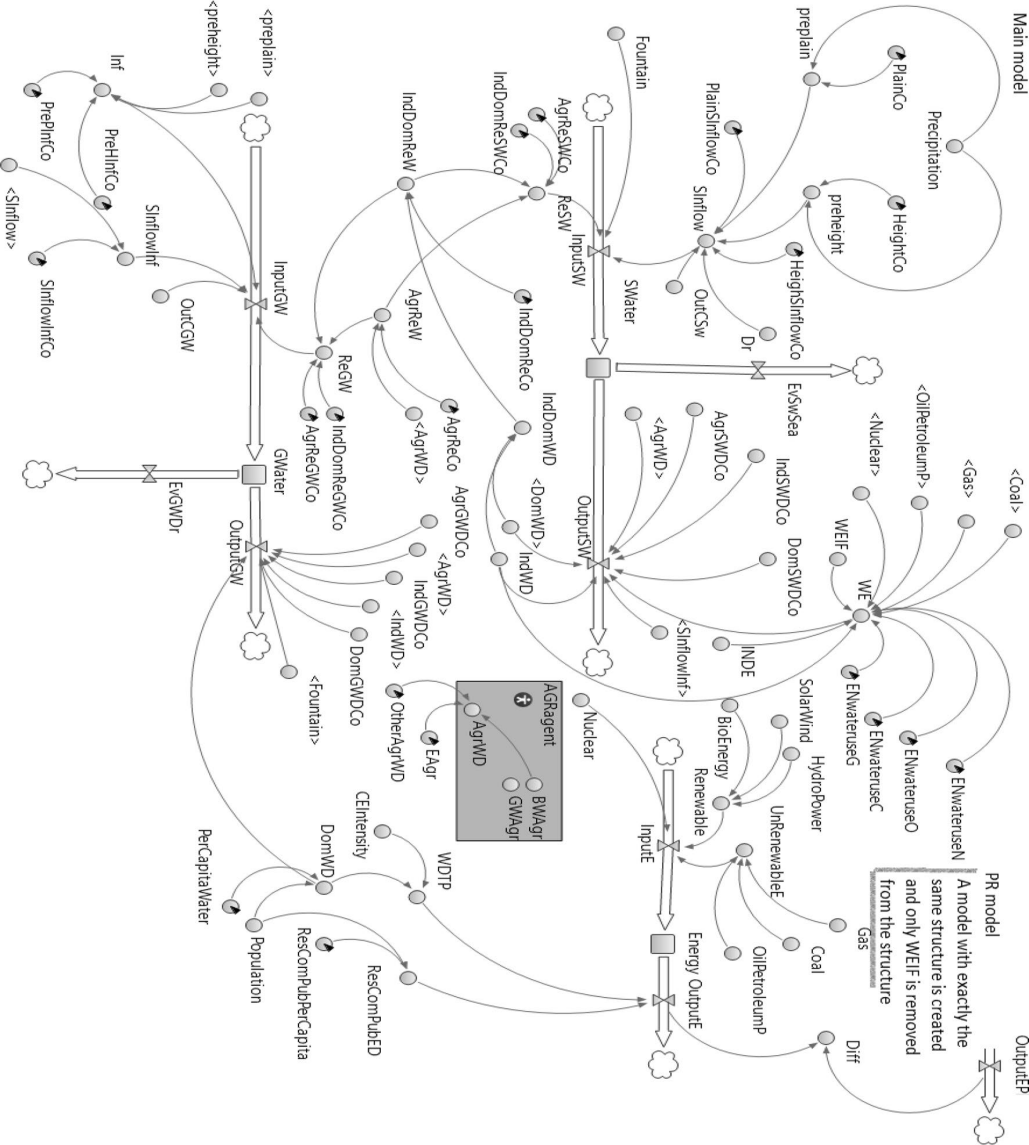
Flow diagram of the WFE Nexus system.

This work formulates a general framework of the WFE Nexus at the national level, which includes all pertinent interactions between water, food, and energy sources and demands. Figure 1 depicts the feedbacks involving resource availability and consumption. The causal loops of the developed model for national-scale assessment are shown in Fig. 2. The model depicted in Fig. 2 proposes reducing consumption to reduce the water crisis to the extent possible. By reducing water use and pollution the environmental water requirement can be reduced, thus alleviating the water crisis. This paper’s objective is sustainable management by reducing per capita water use (in the residential section) and per capita energy use (in the domestic, public, and commercial section). The WFE nexus is modeled as a dynamic system for demand management applied to the stocks of energy, surface water, and groundwater resources to calculate their input and output rates (flows) at the national level while providing for environmental flow requirements (Fig. 3). The national modeling approach is of the lumped type, meaning that inputs and outputs to the stocks of water and energy represent totals over an entire country (in the case study, Iran); therefore, the models does not consider intra-country regional variations. The units of water resources and energy resources are expressed in cubic meters and MWh, respectively.

### Balance of water resources

The study of water exchanges in a country is based on the law of conservation of matter. The following sections present calculations pertinent to the annual balance of surface and groundwater resources.

#### Surface water resources

The national runoff generated in a country’s high-elevation areas (or high terrain) and low-elevation areas (plains) is quantified with the following equations:

1 $${preheight}_{t}=HeightCo\times {Precipitation}_{t}$$

in which ${preheight}_{t}$ = volume of precipitation that falls in high-elevation areas during period *t, *$HeightCo$* =* the percentage of total precipitation that falls in high-elevation areas, and ${Precipitation}_{t}$ = volume of precipitation during period *t*.

2 $${preplain}_{t}=PlainCo\times {Precipitation}_{t}$$

in which ${preplain}_{t}$ = volume of precipitation that falls in the plains during period *t*, and $PlainCo$ = the percentage of total precipitation that falls in plains (low elevation areas).

3 $${SInflow}_{t}=HeighSInflowCo\times {preheight}_{t}+PlainSInflowCo\times {preplain}_{t}+{OutCSW}_{t}+{Dr}_{t}$$

in which ${SInflow}_{t}$ = the total volume of surface flows during period *t*, $HeighSInflowCo$ = the runoff coefficient in high-elevation areas, $PlainSInflowCo$ = the runoff coefficient in the plains, ${OutCSW}_{t}$ = the difference between the volume of surface inflow and outflow through a country’s border during period *t*; and ${Dr}_{t}$ = the flow of groundwater resources to surface water resources (i.e., baseflow) during period *t*.

It is possible to calculate the water use after calculating the annual surface water originating by precipitation. Some of the water use by the agricultural, industrial, and municipal sectors becomes return flows. Equations (4) through (9) show how to calculate the surface water use and the water return flows to the surface water sources.

4 $${DomWD}_{t}={Population}_{t}\times PerCapitaWater\times 365$$

in which ${DomWD}_{t}$ = the volume of water use in the municipal sector during period *t*, ${Population}_{t}$ = the population of the country during period *t*, and $PerCapitaWater$ = per capita drinking water use (cubic meters per person per day).

5 $${IndDomWD}_{t}={DomWD}_{t}+{IndWD}_{t}$$

in which ${IndDomWD}_{t}$ = the volume of water use in the municipal and industrial sectors during period *t,* and ${IndWD}_{t}$ = the volume of water use in the industrial sector during period *t*.

**Figure 4:**
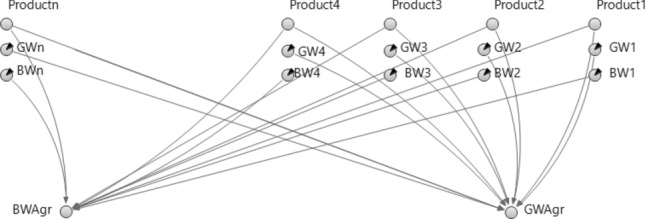
Agricultural subsystem modeled in the AGR agent (shows how to calculate the blue and gray water footprints of agricultural products).

The water use by the agricultural sector accounts for the water footprint of agricultural products, which measures their water use per mass of produce, and adjusting the water use by including water losses and agricultural return flows. A separate sub-agent (AGR agent) is introduced to perform the calculations related to the agricultural sector to simplify the dynamic-system model (main model), and the required outputs (BWAgr, GWAgr) of the dynamic system model are called by the agent in the main model (see Figs. 3 and 4). The BWAgr is given by the expression within parentheses in Eq. (6).

6 $${AgrWD}_{t}=\left(\sum_{i\in A}{BW}_{i}\times {Product}_{i,t}\right)\times \frac{1}{{E}_{Agr}}+OtherAgrWD$$

in which ${AgrWD}_{t}$ = the volume of agricultural water use during period *t*, ${BW}_{i}$ = blue water footprint of agricultural product *i* (cubic meters per ton), ${Product}_{i,t}$ = the amount of production of agricultural product *i* during period *t* (tons), ${E}_{Agr}$ = the overall irrigation efficiency, $OtherAgrWD$ = the volume of water consumed by agricultural products not included in the set A of agricultural products (in cubic meters). The set A includes those agricultural products with the largest yields and shares of the national food basket.

7 $${AgrReW}_{t}={AgrWD}_{t}\times AgrReCo$$

in which ${AgrReW}_{t}$ = the volume of water returned from agricultural water use during the period *t,* and $AgrReCo$ = the coefficient of water returned from agricultural water use.

8 $${IndDomReW}_{t}={IndDomWD}_{t}\times IndDomReCo$$

in which ${IndDomReW}_{t}$ = the volume of water returned from industrial and municipal water use during period *t,* and $IndDomReCo$ = the coefficient of water returned from industrial and municipal water uses.

9 $${ReSW}_{t}=IndDomReSWCo\times {IndDomReW}_{t}+AgrReSWCo\times {AgrReW}_{t}$$

in which ${ReSW}_{t}$ = the volume of water returned from water uses to surface water resources during period *t*, $IndDomReSWCo$ = the percentage of water returned from municipal and industrial water use to surface water resources, and $AgrReSWCo$ = the percentage of water returned from agricultural water use to surface water resources.

Water is applied to produce energy, and Eqs. (10) through (15) perform the related calculations. The ${WEIF}_{t}$ variable in Eq. (14) is necessary to account for the volume of water saved as a result of the energy savings. A PR model is introduced to account for such water savings (see Fig. 3).

10 $${Diff}_{t} ={OutputE}_{t}-{OutputE}_{t}^{P}$$

in which ${Diff}_{t}$= the difference between the energy used in the main model during period *t* and the energy used in period *t* in the PR model, ${OutputE}_{t}$ = the sum of energy uses during period *t* in the main model (the method of calculating ${OutputE}_{t}$ is described in detail in “Energy uses”), and ${OutputE}_{t}^{P}$ = the sum of energy uses during period *t* in the PR model. Equations (11) and (12) account for the case when energy use exceeds energy production under current conditions, in which case energy exports are reduced. This prevents additional energy production to meet excess demand, and, consequently, there would not be increases in water use.

11 $${Diff}_{t} \le 0\,\,\,{if\,\,func}_{t}=0$$

12 $${Diff}_{t} >0\,\,\,{ if\,\,func}_{t}={Diff}_{t}$$

in which ${ iffunc}_{t}$ = the amount of energy saved during period *t*.

Equation (13) calculates the water required to produce energy:

13 $${{TotalWE}_{t}=Coal}_{t}\times ENwateruseC+{Gas}_{t}\times ENwateruseG+{OilPetroleumP}_{t }\times ENwateruseO+{Nuclear}_{t}\times ENwateruseN+{Elec}_{t}\times ENwateruseE$$

in which ${TotalWE}_{t}$ = the volume of water required to produce the energy demand during period *t*,${Elec}_{t}$ = the amount of electricity production during period *t* (MWh), and $ENwateruseE$ = the water required per unit of energy generated by electricity (cubic meters per MWh), all other terms were previously defined.

Equation (14) calculates the water savings:

14 $${WEIF}_{t}=\sum_{t=1}^{T}\frac{{TotalWE}_{t}}{{OutputE}_{t}^{0}}\times {if\,\,func}_{t}$$

in which ${WEIF}_{t}$= the volume of water saved as a result of the energy saved during period *t*, *T =* the number of periods of simulation (*T =* 5 years).

Part of the water used to produce energy from coal, oil, petroleum products, and nuclear fuel is accounted for in the industrial sector water use. For this reason, the volume of water to produce energy calculated with Eq. (15) is reduced by that part of water already accounted for in the industrial water use to avoid double accounting.

15 $${WE}_{t}={Coal}_{t}\times ENwateruseC+{Gas}_{t}\times ENwateruseG+{OilPetroleumP}_{t }\times ENwateruseO+{Nuclear}_{t}\times ENwateruseN-INDE\times {IndWD}_{t}-{WEIF}_{t}$$

in which ${WE}_{t}$ = the volume of water required to produce different types of energy (except those included in the industrial sector) during period *t*, ${Coal}_{t}$ = the energy produced with coal during period *t* (MWh), $ENwateruseC$ = the water required per unit of energy produced with coal (cubic meters per MWh),${Gas}_{t}$ = the amount of energy produced with natural gas during period *t* (MWh), $ENwateruseG$ = the water required per unit of energy produced with natural gas (cubic meters per MWh), ${OilPetroleumP}_{t}$ = the amount of energy produced with crude oil and other petroleum products during period *t* (MWh), $ENwateruseO$ = the water required per unit of energy produced with crude oil and petroleum products (cubic meters per MWh),${Nuclear}_{t}$ = the amount of nuclear energy produced during period *t* (MWh), $ENwateruseN$ = the water required per unit of nuclear energy produced (cubic meters per MWh), and $INDE$ = the percentage of industrial water use already accounted for in Eq. (5) (which pertains to water used in the coke coal, oil refineries, and nuclear fuel industries).

Part of the discharge of springs enters the surface water sources, and this enters the calculation of the input to the surface water-resources stock in Eq. (16):

16 $${InputSW}_{t}= SInflow+{ReSW}_{t}{+ Fountain}_{t}$$

in which ${InputSW}_{t}$ = the volume of inflow water to surface water sources during period *t,* and ${Fountain}_{t}$ = discharge of springs to surface water sources during period *t*, other terms previously defined.

The output of the surface water resources includes water use and the infiltration of surface water into groundwater, the latter calculated with Eq. (17):

17 $${SInflowInf}_{t}={SInflow}_{t}\times SInflowInfCo$$

in which ${SInflowInf}_{t}$ = the infiltration volume of surface water during period *t,* and $SInflowInfCo$ = the infiltration coefficient of surface water.

The output of the surface water resources stock is calculated using Eq. (18):

18 $${OutputSW}_{t}={AgrSWDCo}_{t}\times {AgrWD}_{t}+{IndSWDCo}_{t}\times {IndWD}_{t}+{DomSWDCo}_{t}\times {DomWD}_{t}+{\mathrm{ WE}}_{t}+{SInflowInf}_{t}-{EvSwSea}_{t}$$

in which ${OutputSW}_{t}$ = the output volume of surface water during period *t*, ${AgrSWDCo}_{t}$ = the percentage of gross agricultural water use from surface water resources during period *t*, ${IndSWDCo}_{t}$ = the percentage of industrial water use from surface water resources during period *t*, ${DomSWDCo}_{t}$= the percentage of gross drinking water consumption from surface water sources during period *t*, and ${EvSwSea}_{t}$ = the total volume of evaporation from surface water plus the discharge of surface water to the sea during period *t*.

The balance of surface water resources is calculated based on Eq. (19):

19 $$SWater\left(t\right)=\underset{{t}_{0}}{\overset{t}{\int }}\left[{InputSW}_{t}\left(S\right)-{OutputSW}_{t}(S)\right]dt+SWater(0)$$

in which $SWater\left(t\right)$ = the stock of surface water resources at time *t*, $SWater(0)$ denotes the stock of surface water at the initial time (*t* = 0).

#### Groundwater resources

Groundwater resources gain water from deep infiltration of precipitation in the plains and elevated areas from (1) inflows from outside of the study area, (2) infiltration from surface flows and return waters. Groundwater output factors also include the discharge of groundwater resources (wells, springs, and aqueducts), groundwater flow that moves outside the study area and evaporation. Infiltration of precipitation in the plains and in high terrain into groundwater resources is calculated with Eq. (20):

20 $${Inf}_{t}=PrePInfCo\times {preplain}_{t}+PreHInfCo\times {preheight}_{t}$$

in which ${Inf}_{t}$ = the volume of water entering groundwater sources through infiltration of precipitation during period *t*, $PrePInfCo$ = the infiltration coefficient of precipitation in the plains, and $PreHInfCo$ = the infiltration coefficient of rainfall in high terrain.

Equation (21) calculates the volume of return water that accrues to groundwater resources:

21 $${ReGW}_{t}=IndDomReGWCo\times {IndDomReW}_{t}+AgrReGWCo\times {AgrReW}_{t}$$

in which ${ReGW}_{t}$ = the volume of water returned from water use that accrues to groundwater resources during period *t*, $IndDomReGWCo$ = the percentage of water returned from municipal and industrial water use accruing to groundwater resources, and $AgrReGWCo$ = the percentage of water returned from agricultural water use accruing to groundwater resources.

The volume of groundwater input is calculated with Eq. (22):

22 $${InputGW}_{t}={Inf}_{t}+{ReGW}_{t}+{SInflowInf}_{t}+{OutCGw }_{t}$$

in which ${InputGW}_{t}$ = the volume of groundwater input during period *t,* and ${OutCGw }_{t}$ = the difference between the volume of groundwater leaving and that entering the country during period *t*.

The volume of groundwater output is calculated with Eq. (23):

23 $${OutputGW}_{t}={AgrGWDCo}_{t}\times {AgrWD}_{t}+IndGWDCo\times {IndWD}_{t}+DomGWDCo\times {DomWD}_{t}+{EvGwDr}_{t}$$

in which ${OutputGW}_{t}$ = the volume of groundwater output during period *t*, ${AgrGWDCo}_{t}$ = the percentage of gross agricultural water use from groundwater resources during period *t*, *IndGWDCo* = the percentage of industrial water use from groundwater resources during period *t*, *DomGWDCo* = the percentage of municipal water use from groundwater resources during period *t*, and ${EvGwDr }_{t}$ = the total volume of evaporation from groundwater plus the drainage of groundwater resources to surface water resources at time *t*.

Equation (24) calculates the annual balance of groundwater resources:

24 $$GWater\left(t\right)=\underset{{t}_{0}}{\overset{t}{\int }}\left[{InputGW}_{t}\left(S\right)-{OutputGW}_{t}\left(S\right)\right]dt+GWater(0)$$

in which *GWater*(*t*) = the groundwater resources stock at time *t*, $GWater(0)$ denotes the stock of groundwater at the initial time (*t* = 0).

### Energy uses

Energy uses are calculated with Eqs. (25)–(27). The total national energy use includes the agricultural, industrial, transportation, and exports sectors’ energy demands. The energy uses by these sectors do not change during the implementation of the policy, and, consequently do not change the WFE Nexus in that period; therefore, they are not included in the calculations.

25 $${WDTP}_{t}={DomWD}_{t}\times {CEIntensity}_{t}$$

in which ${WDTP}_{t}$ = the energy used in the extraction, transmission, distribution, and treatment of water in the water and wastewater system during period *t,* and ${CEIntensity}_{t}$ = the energy intensity in the extraction, transmission, distribution, and treatment of water in water and wastewater systems during the period *t* (MWh per cubic meter).

26 $${ResComPubED}_{t}=ResComPubPerCapita\times {Population}_{t}$$

in which ${ResComPubED}_{t}$ = the energy use by the domestic, commercial, and public sectors during period *t*, and $ResComPubPerCapita$ = the per capita energy consumption by the domestic, commercial, and public sectors (MWh per person per year).

27 $${OutputE}_{t}={ResComPubED}_{t}+{WDTP}_{t}$$

### Environmental water needs

The gray water footprint is defined as the volume of freshwater that is required to assimilate the load of pollutants based on natural background concentrations and existing ambient water quality standards. The estimation of the gray water footprint associated with discharges from agricultural production is based on the load of nitrogen fertilizers, which are pervasive in agriculture. The gray water footprint in terms of nitrogen concentration has been estimated by Mekonnen and Hoekstra^24,25^, as written in Eq. (28):

28 $${GW}_{t}^{Agr}=\sum_{i\in A}{GW}_{i}\times {Product}_{i,t}$$

in which ${GW}_{t}^{Agr}$= the volume of gray water in the agricultural sector during period *t,* and ${GW}_{i}$ = the volume of gray water associated with the production of one ton of agricultural product *i* (cubic meters per ton)$.$

There are no accurate estimates of the concentrations of pollutants per unit of industrial production, or of the concentration of pollutants in municipal wastewater. Therefore, the conservative dilution factor (DF), which is equal to 1 for untreated returned water from the municipal and industrial sectors, is applied in this work. Equation (29) is a simplified equation of the gray water footprint^26^. The fraction appearing on the right-hand side of Eq. (29) is equal to the DF.

29 $${GW}_{t}^{IndDom}= \frac{{C}_{eff}-{C}_{nat}}{{C}_{max}-{C}_{nat}}\times {IndDomReW}_{t}\times IndDomReUT$$

in which ${GW}_{t}^{IndDom}$ = the gray water footprint of the municipal and industrial sectors during period *t*, ${C}_{eff}$ = the nitrogen concentration in return water (mg/L), ${C}_{nat}$ = the natural concentrations of contaminant in surface water (mg/L), ${C}_{max}$ = the maximum allowable concentration contaminant in surface water (mg/L), and $IndDomReUT$ = the percentage of untreated returned water from the municipal and industrial sectors.

The total gray water footprint is obtained by summing the footprints associated with the municipal/industrial and agricultural sectors:

30 $${TotalGW}_{\mathrm{t}}={GW}_{t}^{IndDom}+{GW}_{t}^{Agr}$$

in which ${TotalGW}_{\mathrm{t}}$ = the volume of gray water from all sectors during period *t*.

This work considers qualitative and quantitative environmental water needs. Equation (31) is used to calculate the total environmental water need. The Tennant method for calculating the riverine environmental flow requirement (or instream flow) stipulates that, based on the conditions of each basin, between 10 to 30% of the average long-term flow of rivers represents the environmental flow requirement^27^. The sum of these requirements across all the basins equals the environmental requirement of the entire region or country. Yet, by providing 10 to 30% of the average long-term flow of rivers the riverine ecosystem barely emerges from critical conditions, and is far from optimal ecologic functioning. The total environmental water need is equal to the sum of the environmental flow requirement plus the volume of water needed to dilute the contaminants entering the surface water sources:

31 $${ENV}_{t}={TotalGW}_{t}+Tennant$$

in which ${ENV}_{t}$ = the environmental flow requirement during period *t*, and *Tennant* = the environmental flow requirement calculated by the Tennant (1976) method.

### The policy evaluation index

The available renewable water is calculated with Eq. (32):

32 $${IN}_{t}={OutCGW }_{t}+ {SInflow }_{t}+{ Inf}_{t}-{EvGwDr}_{t}$$

in which ${IN}_{t}$= the renewable water available before the application of environmental constraints during period *t*.

The volume of manageable water is calculated with Eq. (33):

33 $$REW\left(t\right)=\underset{{t}_{0}}{\overset{t}{\int }}\left[IN\left(t\right)-ENV\left(t\right)\right]dt$$

in which *REW (t)* = the (cumulative) manageable and exploitable renewable water in the period *t-t*_*0*_.

Equation (34) calculates the total water withdrawals by the agricultural, industrial, municipal, and energy production sectors:

34 $${WDW}_{t}={OutputSW }_{t}+ {OutputGW}_{t}- {cheshmeh}_{t}$$

in which ${WDW}_{t}$ = the sum of the withdrawals by the agricultural, industrial, municipal, and energy production sectors during period *t*.

The cumulative water withdrawals are calculated with Eq. (35):

35 $$withd\left(t\right)=\underset{{t}_{0}}{\overset{t}{\int }}WDW\left(t\right)dt$$

in which $withd\left(t\right)$ = the sum of the withdrawals by the agricultural, industrial, municipal and energy production sectors in the horizon *t-t*_*0*_*.*

Equation (36) calculates the water stress index:

36 $${index}_{{t}_{f}}^{MRW}=\frac{withd({t}_{f})}{REW\left({t}_{f}\right)}\times 100$$

in which ${index}_{{t}_{f}}^{MRW}$ = the renewable water stress index at the end of the study period, and ${t}_{f}$ = the period marking the end of the study horizon.

Once the water and energy model is developed it must be calibrated with observational data prior to its use in predictions, as shown below.

## Study area

**Figure 5:**
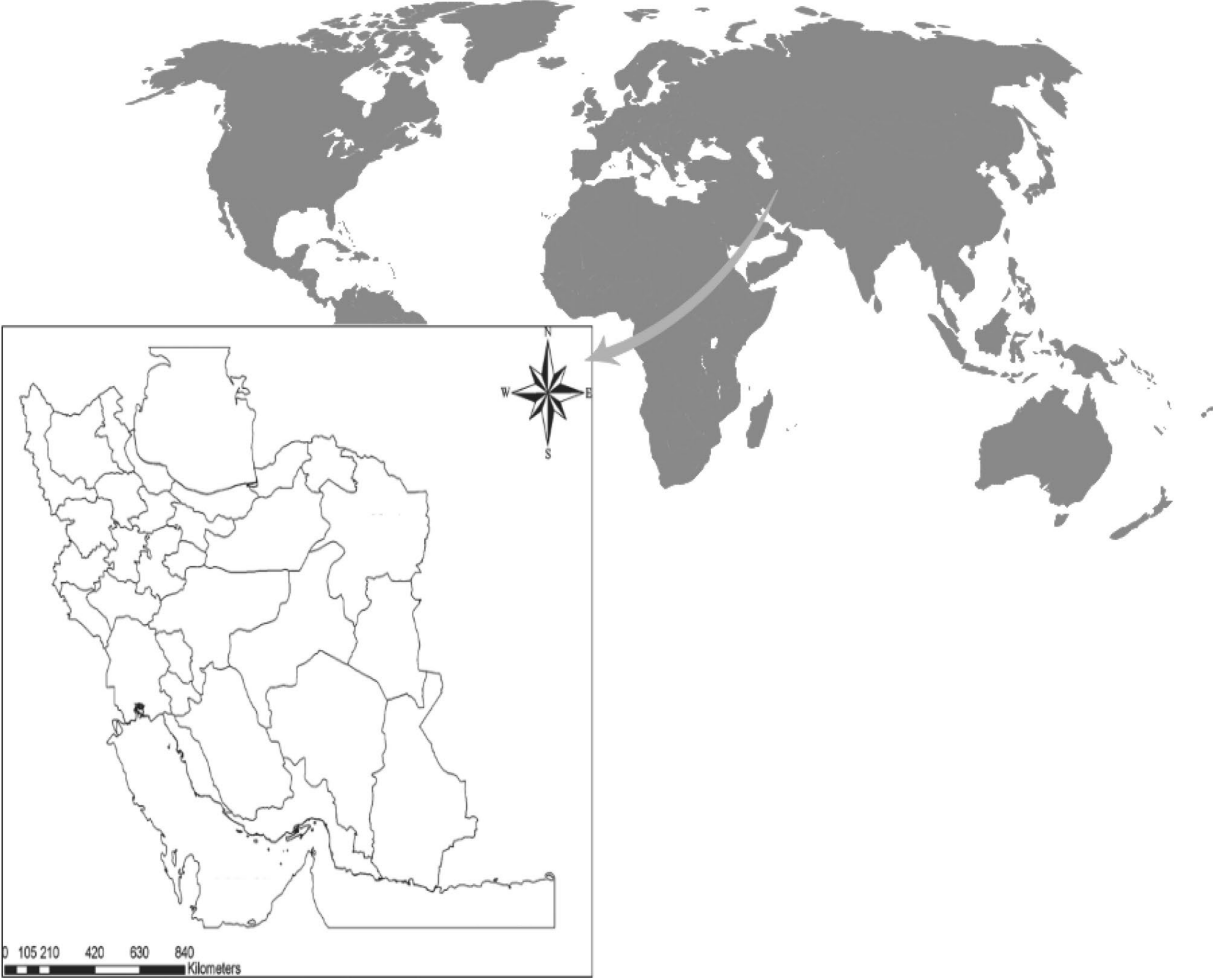
The study area.

Rapid population growth and urban development in the third millennium, especially in developing countries, have significantly increased water demand to meet the needs of expanding populations. Asia accounts for 47% of the global average freshwater per capita, and it accounts for 65% of the world's population; therefore, addressing water supply in these areas is imperative^28^. Western Asia borders southeastern Europe and northeastern Africa. It is seen in Fig. 5 that Iran is located in Western Asia, where rainfall amounts to one third of the world average rainfall, thus being and arid and semi-arid region. Scarce precipitation and rapid population growth mean Iran is grappling with a severe water crisis^29^. Currently, the reported agricultural groundwater withdrawal in all provinces of Iran is more than the total permitted groundwater withdrawal. Significant groundwater level depletion and increasing energy consumption in Iran are important signs of water stress coupled with unsustainable WFE nexus management policies. Continuing the current management paradigm would have critical implications for long-term food and water security^30^.

The water crisis in Iran also harms primary energy production and the utilization of electricity generation capacity. This endangers energy security and socio-economic development at the national level. The negative impact of the water shortage on Iran's electricity sector is due to the dependence of thermal energy and hydropower technologies on water^31^. Also, it is noteworthy that Iran is a leading oil and gas producer. It has the fourth proven oil reserves and the second proven natural gas reserves globally^32^.

Iran is modeled as a lumped region in this work, which means that its national-level water and energy inputs and outputs are simulated without considered intra-country or regional variations over a period of five water years (water year is defined as the 12-month period from October 1st of one year to September 30th of next year) ranging from 2010 through 2014.

The 5-year average values of cross-border water fluxes are as follows:

- The difference between inflows and outflows of surface flows through the land border of the country: five billion cubic meters
- The difference between the inflow and outflow of groundwater flows through the land border of the country: − 269 million cubic meters

This paper’s model is tailored to simulate macro-level policies at the national level, which are pertinent for assessing the WFE security and the provision of environmental flow requirements from a country-wide perspective. Since the model framework of this research is defined on a large scale and in great detail, some of the general information required by the model along with their references are given in the Table 2. The rest of the required information is given in “Agriculture”, “Municipal”, “Industry”, “Water for energy”, “Environment” and “Calibration”.

**Table 2:** General information for the study area and its sources.

Type of data	Population	Precipitation/Groundwater discharge volume/Changes in storage volume of surface water and groundwater resources	Energy generation	Production of agricultural products	Water footprints of various agricultural products
Source	Statistical Center of Iran^50^	Iran's Ministry of Energy^34,51^	Iran's Ministry of Energy^36^	Iran's Ministry of Agriculture Jihad^52^	Iran's Deputy of Infrastructure Research and Production Affairs^53^

### Agriculture

The agricultural sector encompasses 23 agricultural products in this study (sugarcane, sugar beet, lemon, oranges and tangerines, apples, grapes, tomatoes, onions, potatoes, other vegetables, mutton and goat meat, beef, chicken, and eggs, fish, milk, wheat, beans, barley, corn, rice, soybeans and tea), which constitute a major share of the country's agricultural production. The sources of data and their blue and gray footprints are listed in Table 2. The average total irrigation efficiency during the study years equals 40.68%^33^.

### Municipal

The average total volume of urban water withdrawal is estimated at about 8.03 billion cubic meters^34^. The daily per capita municipal water use, which includes all commercial, industrial, domestic and green space consumption, equals 290 liters. Energy is used to extract, transfer, and treat this volume of municipal water. The intensity of this energy use is listed in Table 3^35^. Also, the per capita consumption of domestic, commercial and public energy is 0.77 tons of oil equivalent (8.95 MWh)^36^.

**Table 3:** Energy intensity (kWh/m^3^).

Water year	2010	2011	2012	2013	2014
Energy Intensity	0.50	0.50	0.55	0.55	0.50

### Industry

The industrial sector has the lowest share in water abstraction, which means its water use in all years is the same and equal to the average used in the calculations related to the balance of resources in 2009, i.e., 2.74 billion cubic meters^34^. This volume of water use includes the water uses by the coke industries, oil refining, and production of nuclear fuel, and is approximately equal to 5.4% of the total gross consumption of the industrial sector^37^. This amount of water use must be deducted from the calculations of the water required for the production of various energies.

### Water for energy

Major uses of water for primary energy production include water required for drilling, well completion, injection into reservoirs to increase its recovery factor, extraction of oil sands, refining and processing of primary energy carriers in crude oil and petroleum products. Washing to increase the quality of coal and transportation of coal slurry over long distances and open cycle cooling in power plants also require water. The main source of plant fuels, which are a subset of combustible renewable energy, in Iran is wood from forest trees whose water issues from green water and agricultural products that are neither cultivated nor irrigated for the purpose of energy generation. Therefore, this wood water is not included in the calculation of water used for energy generation. The water used in hydropower plants includes evaporation from the reservoirs, which is considered in the balance of water resources. Table 4 lists the amounts of water required to produce energy (in other words, the water footprint for the production of different types of energy), which varies according to the technologies used in each sector^38^.

**Table 4:** Water required for the production of various types of energy in Iran.

Type of energy	Crude oil and petroleum products	Coal	Natural gas	Electricity
Required water based on production unit (m^3^ per GJ)	1.058	0.164	0.109	0.106

### Environment

The environmental flow requirements or water needs of the country are estimated as the sum of the environmental needs of all its second-degree basins, which were calculated independently and according to the historical statistics and their ecologic conditions. These calculations assume the basins are in relatively good condition (thus the environmental flow requirement ranges from 10 to 30% of the average annual river discharge). The volume of planned surface water resources for environmental use and flow stability of the second-degree basins is estimated at 10,777 million cubic meters^39^. It is necessary to consider wastewater pollutants in estimating the environmental water needs. Therefore, gray water is estimated as explained in the “Proposed methodology and model structure” section. To calculate the gray water of industrial and drinking water consumption it is necessary to know the amount of treated wastewater. 23% of the wastewater produced in the municipal water sector of Iran is treated, but in the industrial sector studies indicate that less than 30% of the industrial wastewater has an efficient treatment system^40^. It is herein estimated that 23% of the total wastewater produced in the industrial and municipal water sectors is treated.

## Calibration

The historic water, gray water, and energy uses were used to calibrate the simulation model herein developed. The percentages of groundwater supply for the agricultural, municipal, and industrial sectors obtained after model calibration are listed in Table 5. The reasons for the decline in the general trend of the percentages of groundwater supply to the municipal and agricultural sectors are illegal withdrawals, installation of illegal wells, and lack of accurate monitoring of water, which have led to a decrease in the volume and quality of groundwater, to landslides in several areas, and to a significant decline in well discharge. Therefore, despite the increase in the number of wells and in water use the percentage of groundwater supply has not risen in recent years. Regulations for controlling illegal wells, especially in the agricultural sector, have proven ineffective. The average annual percentages of water withdrawals from groundwater sources to meet the agricultural, municipal, and industrial water demands are 56.7, 66 and 44%, respectively. This work’s simulation model applied estimated percentages for each year separately, rather than the average annual percentages.

**Table 5:** Variables obtained from calibration.

Water year	Percentage of water supply to sectors from groundwater sources			EvGwDr (m^3^)	EvSwSea (m^3^)
	Agriculture	Industry	Municipal		
2010	63	40	70	1,761,364,343	35,512,000,000
2011	58	47	69	3,674,106,054	35,015,600,000
2012	57	45	66	11,381,759,852	50,826,800,000
2013	54	44	62	13,422,769,751	40,830,800,000
2014	52	46	63	8,470,000,000	26,799,000,000

The simulated model was calibrated after determining the surface water and groundwater uses. Specifically, the model inputs and the residual outputs from surface and groundwater resources were calibrated. The return water coefficients from different uses, the percentage of rainfall in plains and in high terrain, the runoff coefficients in plains and in high terrain, the infiltration coefficients in plains and high terrain, the infiltration coefficients of surface water resources, the sum of evaporation from surface water sources, the output of surface water to the seas, the sum of evaporation from groundwater sources, and the drainage from groundwater sources to surface water sources were calibrated from data extracted from (i) the general water balance in the second-degree catchments of the country, (ii) long-term average data ending in the water year 2010, and (iii) the comprehensive water plan of the country, which were compared with observational data, and by simulation of surface water and groundwater resources in with the calibration tool software and by trial and error. The calibration results are listed in Tables 5 and 6. The predictive skill of the calibrated model was ascertained or tested with various statistical criteria based on observational data. The coefficient R^2^, the Nash-Sutcliffe efficiency (NSE), and the root mean squared deviations (RMSE)-observations standard deviation ratio (RSR) for changes in surface water storage volume were equal 0.99, 0.99 and 0.03, and the same coefficients for changes in groundwater storage volume were equal 0.99, 0.99, and 0.02 thus demonstrating the model’s accurate predictive skill^41^.

**Table 6:** Parameters obtained from calibration.

AgrRe SW Co	IndDomReSW Co	IndDomRe Co	AgrRe Co	PlainSInflow Co	HeighSInflow Co	Plain Co	High-terrain Co
0.09	0.2	0.73	0.28	0.2	0.065	0.7	0.3
IndDomReGW Co	AgrReGW Co		PreHInf Co		PrePInf Co	SInflowInf Co	
0.71	0.91		0.878		0.075	0.128	

## Results and discussion

Water scarcity in Iran is one of the main factors limiting economic development. Measures have been taken in the country concerning water supply for the agricultural, urban, and industrial sectors; yet, less attention has been paid to water use or water demand management. Several instruments for urban water-use management are reduction of Non-Revenue Water (NRW), maintenance of water distribution and storage systems, improvement of residential piping systems, deployment of water-reducing devices, water pricing and education, and deployment of water-efficient landscape irrigation. Per capita residential water use (drinking water) in Iran is approximately 290 liters per person per day, which compares with 130 to 170 liters per day in the United Kingdom^42^, and about 175 liters per person per day in Spain according to its Instituto Nacional de Estadística. Twort et al.^43^ reported a survey of daily per capita residential water ranging from 90 to 150 liters per person in areas with limited water resources. Evidently the daily per capita water use in Iran is relatively high compared to other countries. This paper’s simulation model considers a reduction of the capita use to 150 liters per person per day (maximum acceptable per capita for areas with limited water resources). Moreover, the simulation model considers reducing per capita domestic, public, and commercial energy use to 4.652 MWh per person per year (which is the world average per capita use)^36^. Meanwhile, the Ministry of Energy proposes to save energy in Iran by targeting energy subsidies, reducing losses in electricity distribution companies, as well as replacing old electrical appliances (such as refrigerators) with high-efficiency appliances and modifying the pattern of household consumption. It should be noted that a significant percentage of this reduction in per capita consumption can occur as a result of public education and reform of consumption patterns. But a percentage of these changes also require investment to repair or replace the facility. Realistically, no commercial sector can survive for long if it loses a significant portion of its marketable product, but this is exactly what happens in a large part of the water and electricity systems^44^. For example, if it is assumed that high operating costs and high trade losses do not allow investment to be made in reducing NRW, the high value of NRW will increase slowly but steadily. As a result, it leads to investment in the area related to additional water production and distribution facilities to meet the growing need, and consequently the operating cost increases, and this cycle continues and the economic and social losses intensify. But if there is a continuous investment in NRW management in such a situation, NRW will be reduced, or kept low. As a result, efficient operation leads to a minimum cost of water production and a maximum revenue, and the profits can be used to fund the current NRW management program^44^.

The dynamic system model developed to simulate the current situation as well as the per capita reduction scenario in this study calculated the cumulative balance of water resources through groundwater resources ($GWater$), and surface water resources ($SWater$) stocks. Energy use changes are available by comparing energy output under current situation with the desired scenario. Also, an agent (AGR) is designed for the agricultural sector, in which the agricultural blue and gray water requirement are calculated.

**Figure 6:**
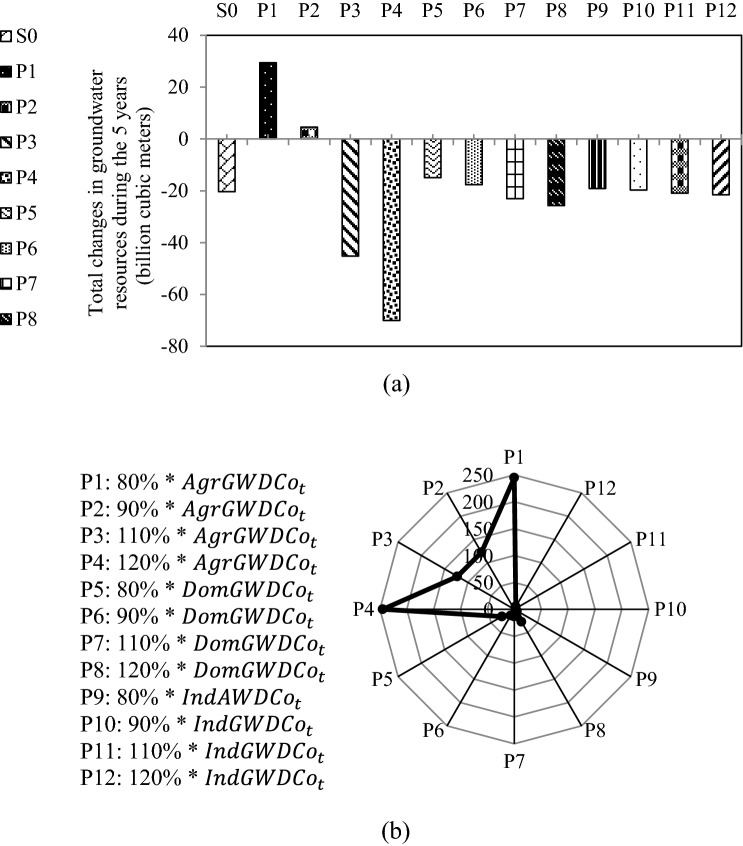
Sensitivity analysis: Impact of changes in the water supply coefficient from groundwater resources in different sectors on the balance of groundwater resources (**a**) In volume, and (**b**) In percentage.

The water uses in the agricultural and industrial sectors are included in the WFE model equations that are used for simulating the surface and groundwater balances. Figure 6 shows the sensitivity of groundwater resources to changes to the water supply coefficient from groundwater resources in different sectors. The results show that the balance of groundwater resources has the highest sensitivity to the coefficient related to the agricultural sector. The fact that agriculture has the largest share in the withdrawal and use of water resources explains this sensitivity. The most negligible impact is related to the coefficient of the industrial sector. Changing this coefficient by 10% changes the cumulative groundwater balance by about 3%, and the sensitivity is more significant for the change in the cumulative surface water balance (with a change of 30%). The difference between these sensitivities is explained by the lower cumulative balance of surface resources compared to that of groundwater resources. Another noteworthy point is that the opposite trend calculated for the changes made in the volume of groundwater resources occurs with respect to the volume of surface water resources. The reason for this is that the total water needs are met with two sources, i.e., surface water and groundwater.

This model features good simulation accuracy due to its high level of detail, and the calibration results (R^2^ = 0.99, NSE = 0.99, and RSR = 0.02–0.03) confirm its accuracy. It is seen in Table 7 that reducing the daily per capita water use to 150 liters per person per day saves water directly, but also indirectly by reducing the water required to produce energy. The average of indirect water-use reduction in the time period studied in this work is equal to 4,596,068 cubic meters. Also, reducing the per capita use of domestic, public, and commercial energy to 4.652 MWh per person per year lowers the total energy use, and, consequently, saves water that would otherwise be used for energy generation. The average of the indirect water savings in this instance equals 744,296,840 cubic meters.

**Table 7:** Volume of water saved indirectly by reducing energy per capita use in the municipal water and domestic, public and commercial energy sector (cubic meters).

Water year	2010	2011	2012	2013	2014
Reducing per capita municipal water use	4,311,083	4,364,549	5,302,374	4,473,430	4,528,903
Reducing per capita domestic, public and commercial energy use	7.26E+08	7.35E+08	7.44E+08	7.53E+08	7.63E+08

**Figure 7:**
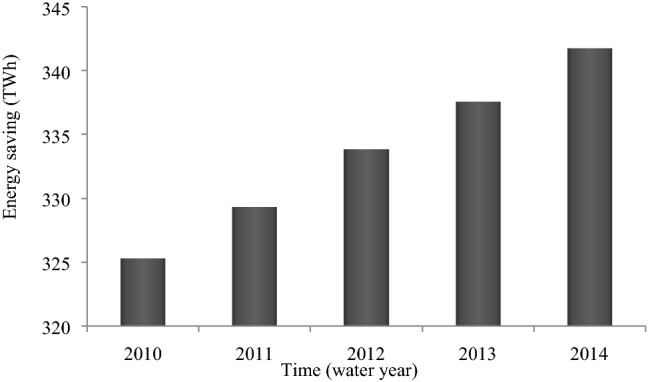
Energy savings from water and energy per capita reductions.

The energy saved (due to the implementation of per capita consumption reduction policies) is in some instanced exported to other countries, generating revenue. In this case the amount of saved water is reduced due to the proportion of saved energy that is allocated to the export sector, because water is used to extract, produce and transfer various types of export fuels. Figure 7 shows the energy saved due to the implementation of the policy of per capita water and energy reductions. which in addition to its effect on reducing water consumption, has a direct impact on reducing greenhouse gas emissions, and it maintain energy reserves to meet future demand. One of the reasons for the upward trend in energy savings is that the population has risen. The energy scenario applies to per capita energy use, and it is therefore directly related to population.

**Figure 8:**
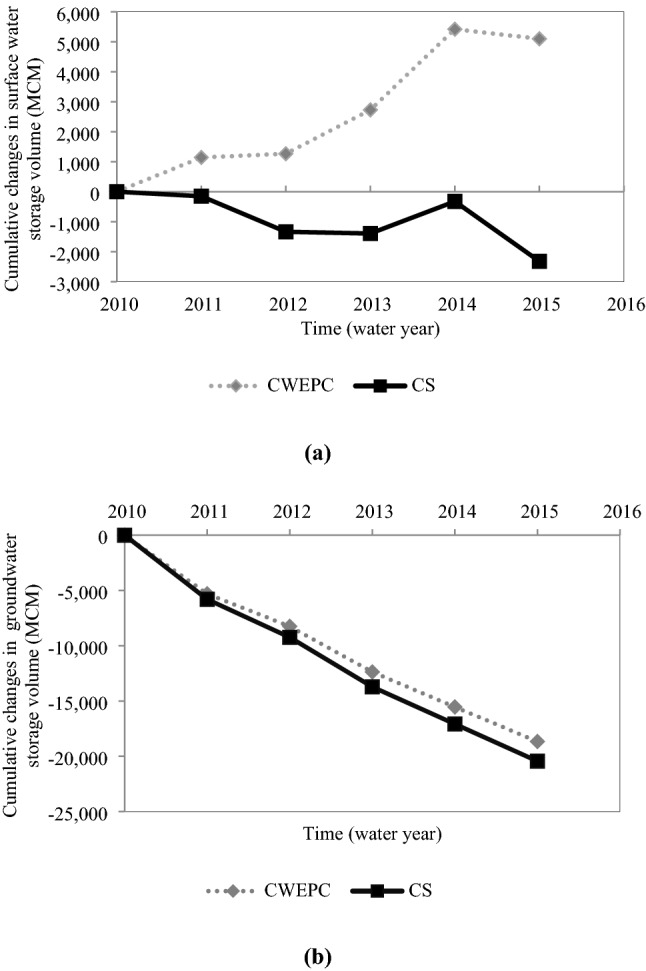
Changes in the cumulative volume of water resources (**a**) Surface water, and (**b**) Groundwater after water and energy per capita reductions are implemented (*CS* current situation, and *CWEPC* changed per capita water and energy uses). MCM = 10^6^ m^3^.

Figure 8 depicts the changes in the cumulative volume of water resources. It is seen in Fig. 8 that the cumulative balance of surface water resources becomes positive as a result of the implementation of the reductions policy. All years, except for the last water year, would experience increasing volumes of surface water resources. This reduction in the volume of surface water resources was due to low rainfall in last water year. This reduction in volume was much less than the current situation. The model projections indicate reductions in surface and groundwater use equal to 7,423 and 1,780 million cubic meters, respectively, during the five-year study period. About 40% of the water saving is related to reduced energy generation, and the saved water is from surface water sources.

Under the current situation, i.e., without per capita reductions, the MRWI at the end of the study period would be 235 %, which, as expected, indicates the stress caused by reliance on non-renewable water sources, and stress in terms of declining water quality for aquatic ecosystems. The MRWI is reduced to 214 % under the per capita reductions policy.

## Concluding remarks

The results of applying the per capita reduction policy in Iran show that despite the small share of residential water and domestic, public, and commercial energy consumption in total water resources consumption, by reducing them, significant changes can be made in the water resources situation. The implementation of the water and energy per capita consumption reduction policy would be costly at the beginning of its implementation in cases when there is a need to modernize the country’s infrastructure to achieve water and energy savings; yet, investments made to implement the policy would provide long-term benefits, among them meeting future water and energy demands and improving environmental quality. This paper’s scenario simulation shows that about 40% of the total deficit of water reservoirs and 9% of the deficit of groundwater reservoirs in the study period are compensated, while reducing the MRWI by 21%. The reduction of the MRWI is the result of reducing water use and pollutant discharge to water bodies through return water.

Future research to further this work’s WFE simulation model may include:

1. Gray water of the industrial and municipal water sectors should be calculated according to the exact volume of returned water produced by each of these sectors and accounting for the concentration of pollutants in the return water.
2. Include the greenhouse gases emission produced in the WFE nexus to improve the environmental assessments.
3. Estimate hydrologic trends and consumption trends to project WFE feedbacks and to project solutions to meet the needs of future generations.
4. Calculate the water and energy uses of each industrial sub-sector, and calculate the total energy and water uses of the industrial sector.
5. Consider comprehensively the energy sector, its components and inputs/outputs to fully account for the feedbacks that exist in the WFE nexus. Separating the types of energy and the amounts of production and use of each type of energy carrier would result in more accurate estimates of the water required for energy production and would facilitate the evaluation of macroeconomic policies.
6. Estimating per capita water use in large cities because of the variability of the per capita use across cities featuring variable urbanization and industrial activity. This refinement would improve water use projections and future water policies.

## Data Availability

All of the required data have been presented in this article.
